# Caspase-11 and caspase-1 differentially modulate actin polymerization via RhoA and Slingshot proteins to promote bacterial clearance

**DOI:** 10.1038/srep18479

**Published:** 2015-12-21

**Authors:** Kyle Caution, Mikhail A. Gavrilin, Mia Tazi, Apurva Kanneganti, Daniel Layman, Sheshadri Hoque, Kathrin Krause, Amal O. Amer

**Affiliations:** 1 Department of Microbial Infection and Immunity, Center for Microbial Interface Biology, Columbus OH 43210; 2 Dorothy M. Davis Heart and Lung Research Institute, and The Ohio State University, Columbus OH 43210.

## Abstract

Inflammasomes are multiprotein complexes that include members of the NOD-like receptor family and caspase-1. Caspase-1 is required for the fusion of the *Legionella* vacuole with lysosomes. Caspase-11, independently of the inflammasome, also promotes phagolysosomal fusion. However, it is unclear how these proteases alter intracellular trafficking. Here, we show that caspase-11 and caspase-1 function in opposing manners to phosphorylate and dephosphorylate cofilin, respectively upon infection with *Legionella*. Caspase-11 targets cofilin via the RhoA GTPase, whereas caspase-1 engages the Slingshot phosphatase. The absence of either caspase-11 or caspase-1 maintains actin in the polymerized or depolymerized form, respectively and averts the fusion of pathogen-containing vacuoles with lysosomes. Therefore, caspase-11 and caspase-1 converge on the actin machinery with opposing effects to promote vesicular trafficking.

Actin filaments play a role in cell cycle, cellular motility, and proper vesicle transport, and are critical for providing structure and subcellular organization^1^. In addition, trafficking and fusion of the phagosome with the lysosome is a key host defense mechanism modulated by the actin machinery^2^. In order for fusion to occur, actin nucleates and polymerizes around the membranes of phagosomes providing a directional track to interact and fuse with lysosomes^3^. The dynamic process of actin polymerization and depolymerization is regulated by the phosphorylation status of cofilin^4^. Cofilin activity is inhibited by phosphorylation of the serine residue at position 3, which is mediated by the Rho small GTPase family: RhoA, Rac, or Cdc42. Activation of RhoA, Rac, and Cdc42 in hematopoietic cells leads to the polymerization of actin into distinct structures. On the other hand, Slingshot family of protein phosphatases dephosphorylates and reactivates cofilin^5^,^6^. These modifications cycle cofilin from active to inactive states^4^. Whether these GTPase and phosphatases play a role during intracellular infections is unknown. In addition, it is also unclear if their activation is altered by the caspase-1 and caspase-11.

*Legionella pneumophila* (*Legionella*) is a facultative, intracellular pathogen that causes Legionnaire’s disease (LD). The majority of people exposed to *Legionella* remain asymptomatic or suffer only mild self-limiting infection. Cigarette smoking, chronic lung disease, and immunosuppression have been consistently implicated as risk factors^7^,^8^. *Legionella* reaches the alveoli where it encounters and multiplies in human alveolar macrophages at the site of infection, which is crucial to the pathogenesis of LD^9^,^10^,^11^. Notably, human monocytes do not activate caspase-1 and -7 upon *Legionella* infection, allowing bacterial growth^12^,^13^,^14^,^15^. Primary human macrophages and the THP-1 monocytic cell line also do not activate caspase-1 in response to *Legionella*^12^,^13^,^14^.

In contrast, macrophages from C57BL/6 mice (designated wild-type (WT) in the text) restrict *Legionella* replication via activation of caspase-1, caspase-7, and caspase-11 resulting in fusion of the containing phagosome with the lysosome leading to bacterial degradation and growth restriction^14^. In WT mouse macrophages, bacterial flagellin leaks through the type-IV secretion system (T4SS) and is detected within the host cytosol by the NOD-like receptors (NLR) Nlrc4 and Naip5, leading to the activation of caspase-1^16^,^17^,^18^,^19^,^20^,^21^. Independently of the inflammasome complex, caspase-11 is induced and activated upon *Legionella* infection contributing to bacterial clearance by promoting phagosome-lysosome fusion^14^. Accordingly, mouse macrophages lacking any of these factors (e.g. *Nlrc4*^−/−^, *Naip5*^−/−^, *Casp1*^−/−^, *Casp-7*^−/−^, or *Casp11*^−/−^) are permissive to *Legionella* infection^17^,^22^,^23^,^24^. However, the signaling pathway linking caspase-1 and caspase-11 to vesicular trafficking remains obscure.

Here we demonstrate that cofilin loses its basal phosphorylation status early upon *Legionella* infection in WT macrophages. Dephosphorylation of cofilin is accompanied by an increase in the filamentous actin (F-actin) and globular actin (G-actin) ratio and promotion of fusion of the *Legionella*-containing vacuole with the lysosome. We found that caspase-11 is required for the phosphorylation of RhoA and cofilin, whereas caspase-1 promotes the dephosphorylation of cofilin via Slingshot. In WT macrophages, where both caspases are active, the F/G ratio dynamically changes during *Legionella* infection, as it increases within 2 hours of infection and the pathogen-containing vacuole fuses with the lysosome. When caspase-11 or caspase-1 is lacking, such as in their corresponding single knock outs, cofilin remains phosphorylated or dephosphorylated, respectively. The F/G-actin ratio remains unchanged and the fusion of the pathogen-containing vacuole with the lysosome is halted and *Legionella* replicates. Notably, the enzymatic activity of caspase-11 is required for its ability to modulate actin polymerization. Interestingly, the absence of either caspase-11 or caspase-1 does not influence the trafficking of *E. coli*-containing vacuoles. Our results identify new key players downstream of caspase-11 and caspase-1 that modulate the trafficking of phagosomes harboring pathogenic intracellular microbes.

## Results

### The *Legionella*-containing vacuole fails to fuse with the lysosome in macrophages lacking caspase-1

**Figure 1:**
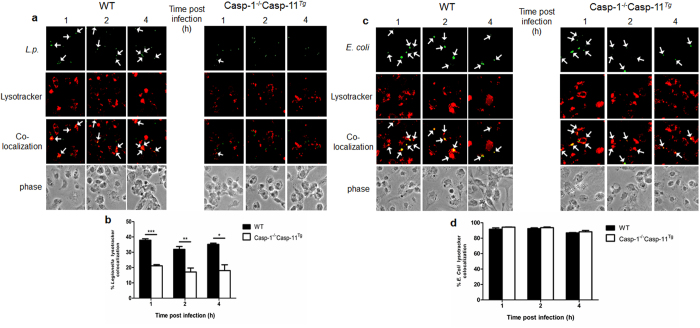
Caspase-1 promotes fusion of the *Legionella* vacuole with lysosome. Wild-type (WT) and Casp-1^−/−^Casp-11^Tg^ macrophages were infected with (**a**) GFP-expressing *Legionella* (*L.p*.) or (**c**) *Escherichia* (*E.*) *coli*. Macrophages were stained with LysoTracker® red that accumulates in acidic compartments. Quantification of colocalization of LysoTracker® with (**b**) GFP-*L.p.* and (**d**) GFP-*E.coli*. Mean percent colocalization ± SEM from three biological replicates are represented. (**b**) significance, indicated by (*), was obtained by performing Student’s unpaired *t*-test. p = 0.0002 is ***, p = 0.01 is **, and p = 0.0113 is *.

It has been previously shown that the *Legionella*-containing vacuole (LCV) fails to fuse with the lysosome in macrophages believed to only lack caspase-1^17^,^22^. However, the caspase-1^−/−^ macrophages used in previous publications also lacked caspase-11^25^,^26^,^27^,^28^,^29^. Thus, it is possible that caspase-11 alone is sufficient to promote phagosome-lysosome fusion, irrespective of caspase-1 expression. To test this hypothesis, we examined the role of caspase-1 using the single caspase-1 knock out (*Casp*-1^−/−^*Casp*-11^Tg^) during *Legionella* infection (a generous gift from Dr. Vishva Dixit, Genetech)^30^. We focused on mimicking early stages of physiological infection, and we used low multiplicity of infection (MOI). First, we evaluated the trafficking of *Legionella* in Casp-1^−/−^Casp-11^Tg^ bone marrow-derived macrophages (BMDMs). Fusion of the LCV with the lysosome was assessed by quantifying the number of GFP-expressing bacteria colocalized with LysoTracker™ red, a dye that stains acidic vacuoles. In WT macrophages, intracellular bacteria readily trafficked to lysosomes (~38%), while in Casp-1^−/−^Casp-11^Tg^ cells, significantly less bacteria (~20%) did so (Fig. 1a,b). Conversely, *Escherichia coli* (*E. coli*) trafficked to the lysosome regardless of caspase-1 expression (~90%) (Fig. 1c,d).

**Figure 2:**
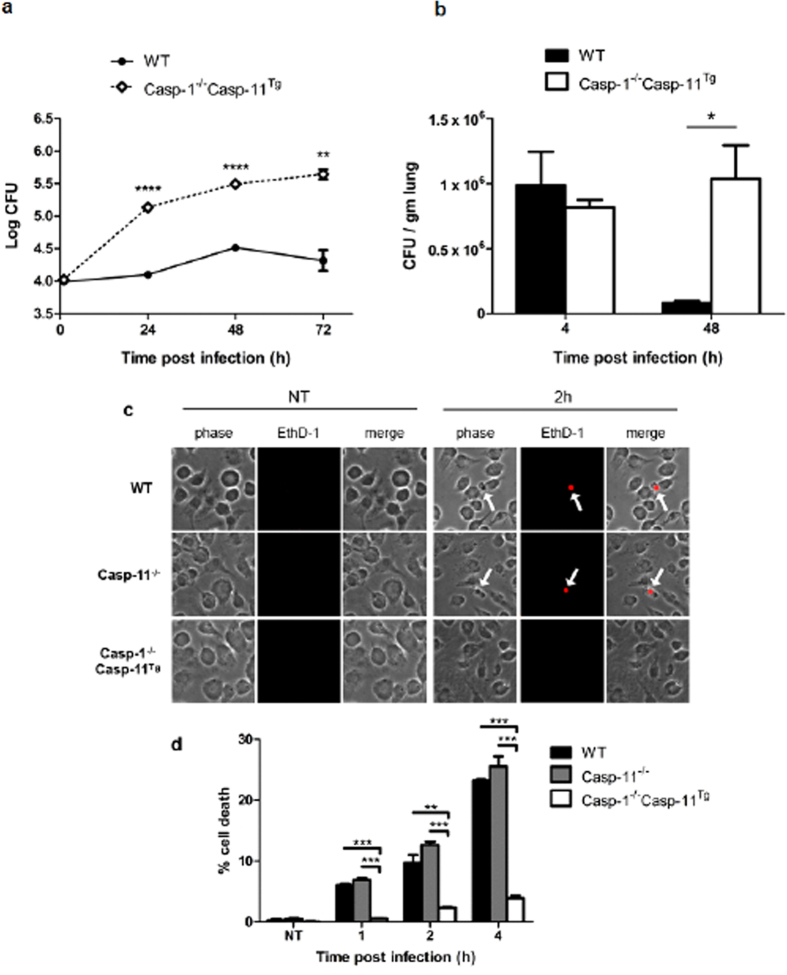
Caspase-1 promotes the restriction of *Legionella in vitro* and *in vivo*. Colony forming unit (CFU) assay of *Legionella* replication in WT and Casp-1^−/−^Casp-11^Tg^ (**a**) *in vitro* after 1, 24, 48, 72 hrs post infection and (**b**) *in vivo* from lungs of WT and Casp-1^−/−^Casp-11^Tg^ mice at 4 and 48 hrs after infection. (**c**) Live/Dead® immunofluorescence assay on WT, Casp-11^−/−^, Casp-1^−/−^Casp-11^Tg^ BMDMs infected (2 hrs) or not (NT) with *Legionella*. (**d**) Quantification of Live/Dead® stain after 1, 2, and 4 hrs after infection and mean percent dead macrophages ± SEM from three biological replicates are represented. (**a**) significance, indicated by (*), was obtained by performing Student’s unpaired *t*-test. 24 hrs p = 0.000098 is ****, 48 hr p = 0.000087 is ****, 72 hr p = 0.0017 is **, respectively. (**b**) 48 hrs p = 0.021 is *. (**d**) significance of the Live/Dead assay was calculated by one-way ANOVA with Student’s *t*-test post-test. *** is p < 0.001 and ** is p < 0.01.

To determine the independent role of caspase-1 in the pathogenesis of Legionnaire’s disease, replication of *Legionella* in macrophages (*in vitro*) and lungs (*in vivo*) of Casp-1^−/−^Casp-11^Tg^ mice was assessed via colony forming units (CFUs). During *in vitro* infection, Casp-1^−/−^Casp-11^Tg^ macrophages exhibited significantly more bacterial replication at 24, 48, and 72 hrs after infection (Fig. 2a). *In vivo*, bacterial loads in the lungs of the Casp-1^−/−^Casp-11^Tg^ mice after 4 hrs of infection were similar, indicating equivalent initial infection doses. After 48 hrs of infection, significantly more *Legionella* CFUs were recovered from lungs of Casp-1^−/−^Casp-11^Tg^ mice compared to that of WT counterparts (Fig. 2b). These results indicate that caspase-1 contributes to the efficient fusion of *Legionella*-containing vacuoles with the lysosome and restriction of the bacteria *in vitro* and *in vivo*.

It has also been reported that caspases promote host cell death during infection in order to restrict bacterial replication^31^,^32^,^33^. Previous studies using the caspase-1/11 double knockout have shown significantly reduced levels of cell death during *Legionella* infection without reference to which caspase is a major determinant in the host cell death process^34^,^35^. To elucidate the singular role of these caspase in cell death, we performed a Live/Dead immunofluorescence assay where dead cells allow ethidium homodimer-1 (EthD-1) to reach their nuclei and stain them red. WT, Casp-11^−/−^, and Casp-1^−/−^Casp-11^Tg^ BMDMs were infected with *Legionella* and then stained with EthD-1. We found that after 1, 2, and 4 hrs infection, Casp-1^−/−^Casp-11^Tg^ macrophages displayed significantly less cell death when compared to WT and Casp-11^−/−^ BMDMs (Fig. 2c,d). There was no significant difference in percentage of cell death between WT and Casp-11^−/−^ macrophages throughout the infection time course. These data indicate that during *Legionella* infection, caspase-1, not caspase-11, is a major determinant in promoting cell death that also contributes to the restriction of the bacterium.

### The F/G actin ratio increases in macrophages upon *Legionella* infection

**Figure 3:**
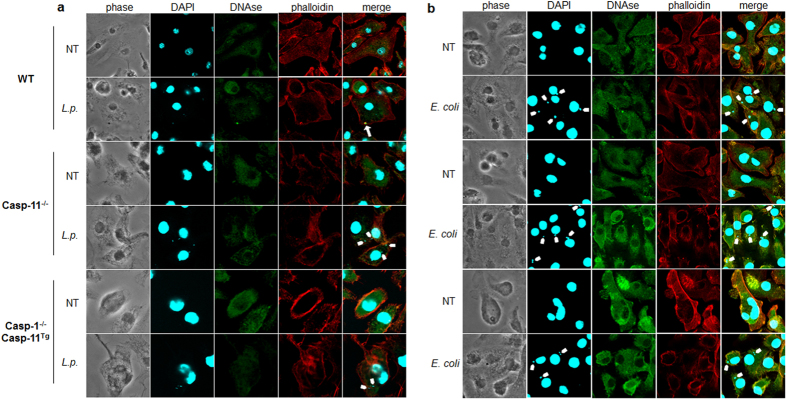
Caspase-11 and caspase-1 promote F-actin polymerization in the vicinity of the *Legionella*-containing vacuole. Confocal analysis of WT, Casp-11^−/−^, and Casp-1^−/−^Casp-11^Tg^ macrophages double-labeled with Alexa Fluor 568 phalloidin (stains F-actin) and Alexa Fluor 488 DNAse I (stains G-actin) after 2 hrs infection with (**a**) *Legionella* (*L.p.*) or (**b**) *Escherichia (E.*) *coli*. F-actin structures in the vicinity of the *Legionella* vacuole in WT macrophages are indicated by white arrow, white tabs indicate their absence. Data are representative of three biological replicates performed independently.

**Figure 4:**
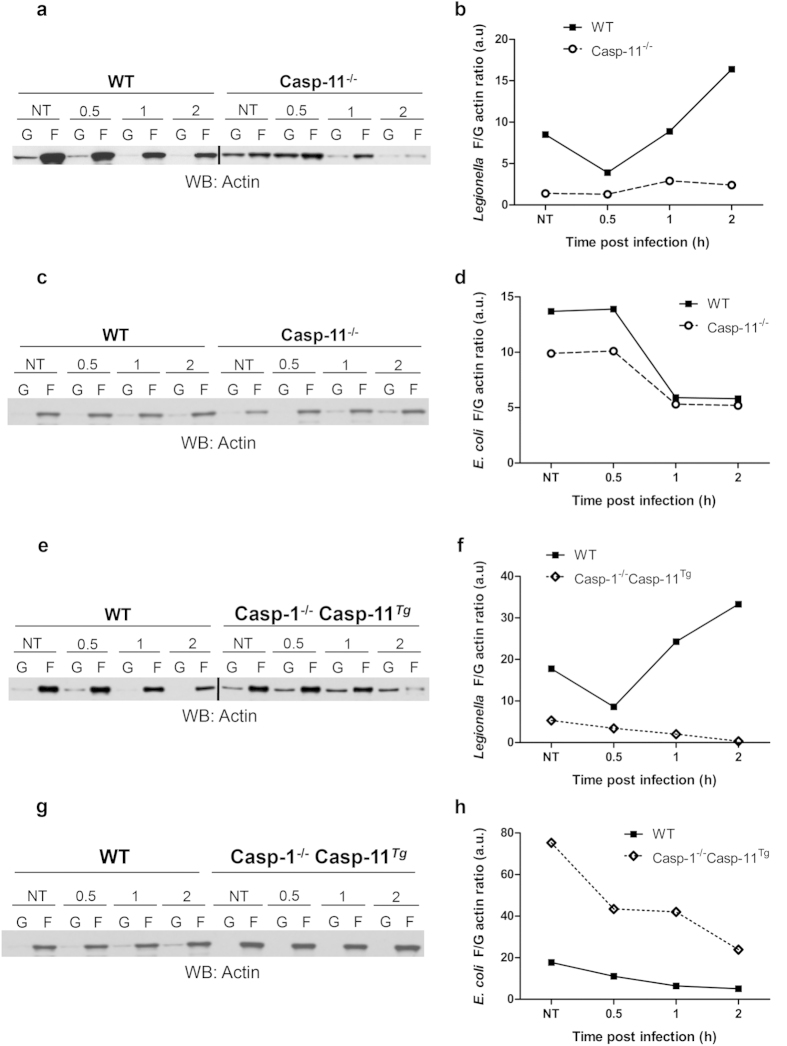
F/G actin ratio is increased in WT macrophages, while Casp-11^−/−^ and Casp-1^−/−^Casp-11^Tg^ cells display less during *Legionella* (*L.p.*) infection. G-actin/F-actin assay in WT and Casp-11^−/−^ macrophages infected with (**a**) *L.p.* and (**c**) infected with *E. coli* for 0.5, 1, and 2 hrs. Dynamic changes in F/G actin ratios were quantified (**b**,**d**). G-actin/F-actin assay of WT and Casp-1^−/−^Casp-11^Tg^ macrophages infected with (**e**) *L.p.* and (**g**) *E. coli* for 0.5, 1, and 2 hrs and changes in F/G ratios were quantified (**f**,**h**). Data are representative of three independent experiments.

The host actin cytoskeleton dynamically changes during chemotaxis, phagocytosis, and vesicle transport^36^. Late endosomes and phagosomes use filamentous (F) actin as a scaffold or track to move towards and efficiently fuse with other endocytic compartments and lysosomes^37^. To enhance our understanding of the role of caspases in vesicular trafficking during *Legionella* infection WT, Casp-11^−/−^, and Casp-1^−/−^ Casp-11^Tg^ BMDMs were used to decipher the singular contribution of caspase-1 to actin modulation^38^. Macrophages were double stained with Alexa Fluor 568 phalloidin to label F-actin structures and Alexa Fluor 488 DNAse I to label unpolymerized G-actin and examined via confocal microscopy. Basally, WT macrophages expressed evenly distributed F-actin structures throughout the cell (Fig. 3a). Comparatively, Casp-11^−/−^ and Casp-1^−/−^Casp-11^Tg^ macrophages displayed F-actin that accumulates at the periphery of the cell, while there is more G-actin throughout the cell body (Fig. 3a). Upon *Legionella* infection of WT macrophages, *F*-actin structures localized in the vicinity of *Legionella*, whereas, in Casp-11^−/−^ and Casp-1^−/−^Casp-11^Tg^ macrophages, the distribution of F-actin remained unchanged (Fig. 3a). Notably, the distribution of F-actin during infection with non-pathogenic *E. coli* remained unchanged in the presence or absence of caspase-11 and caspase-1 (Fig. 3b). Since end point measurements of F-actin do not reflect actin dynamics, we assessed the changes in actin polymerization by measuring the ratio of polymerized (F) and unpolymerized (G) actin throughout 2 hours of infection. WT, Casp-11^−/−^, and Casp-1^−/−^Casp-11^Tg^ macrophages were infected with *Legionella* for 0.5, 1, and 2 hrs, lysed, and soluble (G-actin) and insoluble (F-actin) cytoplasmic fractions were separated and analyzed by western blot. Restrictive WT macrophages exhibited dynamic reduction then increased ratios of F/G actin during *Legionella* infection. Comparatively, in permissive macrophages lacking caspases-11, the F/G ratio remained unchanged throughout infection (Fig. 4a,b). In the absence of caspase-1, the F/G actin ratio slightly decreased throughout the elapsed infection time (Fig. 4e,f). To determine if the modulation of the actin cytoskeleton is similar during pathogenic and non-pathogenic bacterial infection, Casp-11^−/−^ and Casp-1^−/−^Casp-11^Tg^ macrophages were infected with *E. coli.* Vacuoles harboring these non-pathogenic bacteria fuse with the lysosome in the absence or presence of caspase-1 and caspase-11 (Fig. 1c,d). The ratio of F/G actin was assessed throughout infection. WT, Casp-11^−/−^, and Casp-1^−/−^Casp-11^Tg^ cells exhibited steep reduction of the F/G actin ratios irrespective of caspase-1 and caspase-11 (Fig. 4c,d,g,h). Together, these data indicate that alteration of the F/G actin ratio is required for the trafficking of vacuoles harboring intracellular pathogens to lysosomes but not for those carrying non-pathogenic organisms. In addition, these results show that caspase-11 and caspase-1 are both necessary to increase the F/G actin ratio during *Legionella* infection but are dispensable for the trafficking of vacuoles containing non-pathogenic bacteria.

### Caspase-11 is required for the phosphorylation of cofilin, whereas caspase-1 promotes its dephosphorylation

**Figure 5:**
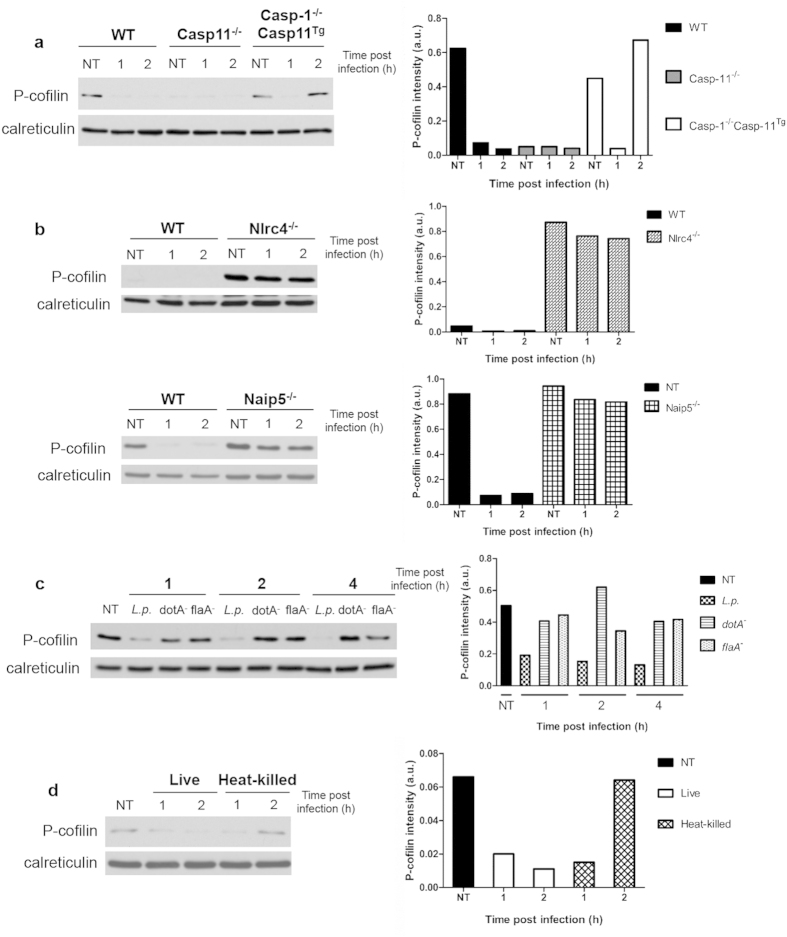
Caspase-11 and caspase-1 differentially regulate the phosphorylation of cofilin. Immunoblot analysis of phospho-cofilin (P-cofilin) after infection with *Legionella* in (**a**) WT, Casp-11^−/−^, and *Casp*-1^−/−^*Casp*-11^Tg^. (**b**) Nlrc4^−/−^ and Naip5^−/−^ macrophages. (**c**) P-cofilin immunoblots of WT cells infected with parental *Legionella* (*L.p*.), the T4SS (*dotA*^*−*^) mutant and flagellin (*flaA*^*−*^) mutant for 1, 2, and 4 hrs. (**d**) Lysates from WT macrophages infected with live and heat-killed *Legionella* analyzed for P-cofilin at 1 and 2 hrs after infection. Blots are representative of at least three independent experiments.

Because caspase-11 and -1 are both required for the restriction of *Legionella* and are linked to the actin cytoskeleton during different cell processes^14^ (Figs 1, 2, 3, 4), we further investigated the signaling pathways that are shared or unique downstream of these caspases. Actin polymerization and depolymerization is regulated by cofilin phosphorylation status^4^,^39^. WT, Casp-11^−/−^, and Casp-1^−/−^Casp-11^Tg^ macrophages were infected with *Legionella* and the phosphorylation of cofilin was determined by western blot. Resting macrophages elicited different patterns of cofilin phosphorylation. While resting WT and Casp-1^−/−^Casp-11^Tg^ macrophages demonstrated the phosphorylated form of cofilin, Casp-11^−/−^ macrophages did not (Fig. 5a). Upon *Legionella* infection, WT macrophages lost the phosphorylation of cofilin within 1 hour infection while Casp-11^−/−^ macrophages failed to alter the status of cofilin phosphorylation throughout infection. On the other hand, in Casp-1^−/−^Casp-11^Tg^ cells, the phosphorylation of cofilin initially decreased, but then increased after 2 hrs of infection (Fig. 5a). Taken together, these data indicate that caspase-11 and caspase-1 play opposing roles in modulating cofilin phosphorylation status during infection, as caspase-11 is required for the phosphorylation of cofilin and caspase-1 is necessary for its dephosphorylation.

### Nlrc4 and Naip5 are required for cofilin dephosphorylation upon *Legionella* infection

The NOD-like receptors (NLRs) Nlrc4 and Naip5 form a multiprotein inflammasome complex that is able to detect intracellular bacteria, triggering canonical pyroptosis and non-pyroptotic clearance of pathogens^16^,^17^,^22^,^40^. Because the inflammatory caspases are activated downstream of Nlrc4 and Naip5 and since caspase-11 and -1 have been shown to modulate the activity of cofilin, we investigated whether Nlrc4 and/or Naip5 are able to control the phosphorylation status of cofilin in response to *Legionella*. WT, Nlrc4^−/−^, and Naip5^−/−^ macrophages were infected and 1 and 2 hrs of infection, both Nlrc4^−/−^ and Naip5^−/−^ cells failed to dephosphorylate cofilin when compared to WT cells (Fig. 5b). Since Nlrc4 and Naip5 detect bacterial flagellin within the host cytosol, we determined if alterations in cofilin phosphorylation are dependent on flagellin. WT macrophages were infected with a T4SS (*dotA*^−^) and a flagellin (*flaA*^*−*^) mutant of *Legionella*. After 1, 2, and 4 hrs of infection, Western blot analysis showed that the *dotA*^−^ and the *flaA*^*−*^ mutants failed to modulate cofilin phosphorylation status when compared to the parental *Legionella* strain (Fig. 5c). To understand whether this process is due to an active process elicited by live bacteria, we infected WT cells with live or heat-killed *Legionella*. Live bacteria dephosphorylated cofilin, while the heat-killed bacteria failed to do so (Fig. 5d). Together, these data indicate that live, flagellated *Legionella* with a competent T4SS dephosphorylates and activates cofilin in a Nlrc4/Naip5/caspase-1 axis-dependent manner.

### The activation of RhoA in response to *Legionella* requires caspase-11

**Figure 6:**
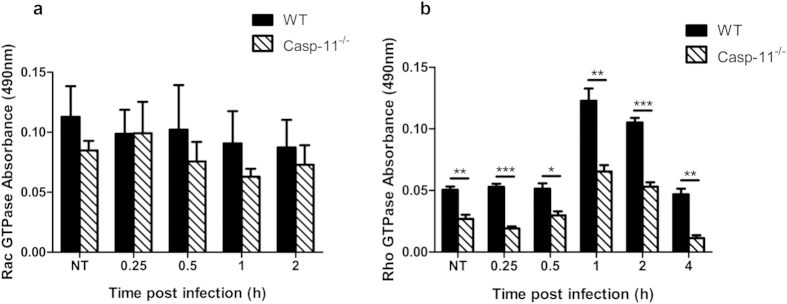
Caspase-11-dependent phosphorylation of cofilin requires the RhoA GTPase activity. Rac GTPase activity (**a**) and RhoA GTPase activity (**b**) from WT and Casp-11^−/−^ macrophage lysates after infection with *Legionella* using specific G-LISA activation assay (Cytoskeleton) 0.25, 0.5, 1, and 2 hrs post infection. All data are representative of at least three independent experiments. G-LISA assays analyzed for significance by Student’s unpaired *t*-test. *** is p < 0.001, ** is p < 0.01, and * is p<0.05.

The phosphorylation of cofilin is driven by the GTPases: RhoA, Rac, and/or Cdc42 through Limk in response to different stimuli^1^. Therefore, we assessed GTPase activity using specific G-LISA Activation Assays (Cytoskeleton). Lysates from WT and Casp-11^−/−^ macrophages displayed similar levels of Rac activation in response to *Legionella* infection, and this was confirmed by Western blot using phospho-Rac/Cdc42 antibodies (Fig. 6a and data not shown). Notably, Casp-11^−/−^ macrophages exhibited significantly lower levels of RhoA activity during *Legionella* infection when compared to WT cells (Fig. 6b). These data indicate that upon *Legionella* infection, caspase-11 is required for RhoA GTPase activation, which is then accompanied by phosphorylation of cofilin.

### Caspase-1 is required for the activity of the phosphatase Slingshot during *Legionella* infection

**Figure 7:**
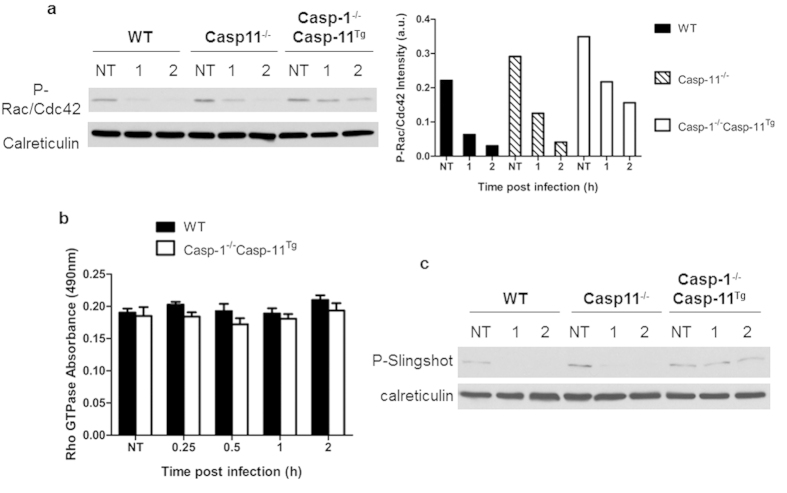
Slingshot-mediated cofilin dephosphorylation in response to *Legionella* infection is dependent on caspase-1. WT and Casp-1^−/−^Casp-11^Tg^ macrophages assayed for activity of (**a**) Rac/Cdc42 by Western blot analysis using phospho-Rac/Cdc42 antibodies 1 and 2 hrs post infection, (**b**) RhoA GTPase using specific G-LISA activation 0.25, 0.5, 1, and 2 hrs after infection, and (**c**) Slingshot by immunoblot using phospho-Slingshot specific antibody after 1 and 2 hrs infection with *Legionella*. Data are representative of at least three independent experiments. (**b**) Student’s unpaired *t*-test was performed to determine significance, time points exhibited no significance (NS).

Cofilin is dephosphorylated via the phosphatase Slingshot^41^. To determine if caspase-1 is required for the dephosphorylation of cofilin in response to *Legionella* infection by halting the activity of kinases or promoting the activity of phosphatases that converge on cofilin, we first assessed Rac1 and Cdc42 activation. WT and Casp-1^−/−^Casp-11^Tg^ macrophages were infected with *Legionella* then analyzed by western blot using specific phospho-antibodies. Casp-1^−/−^Casp-11^Tg^ macrophages displayed a similar trend of decreased levels of phospho-Rac1/Cdc42, compared to WT and Casp-11^−/−^ counterparts indicating that modulation of Rac and/or Cdc42 is independent of caspase-11 and caspase-1 (Fig. 7a). Next, we assessed the activity of RhoA using a G-LISA assay specific for its active form (GTP bound). The activity of RhoA in WT and Casp-1^−/−^Casp-11^Tg^ macrophages did not differ upon *Legionella* infection, indicating that the activity of RhoA was dependent on caspase-11 and not caspase-1 (Fig. 7b). We next determined if the activation of the phosphatase protein Slingshot is altered by the lack of caspase-1. The activation of the Slingshot protein is determined by its phosphorylation status as phosphorylation of Slingshot inhibits phosphatase activity while dephosphorylation activates Slingshot^42^. Using a specific antibody against the phosphorylated Ser-978, Fig. 7c shows that Slingshot is dephosphorylated during *Legionella* infection in WT and Casp-11^−/−^ macrophages. Conversely, in Casp-1^−/−^Casp-11^Tg^ macrophages, Slingshot remained phosphorylated (inactivated) throughout the infection (Fig. 7c). Together, this data demonstrate that the activation of Slingshot requires the presence of caspase-1 and not caspase-11.

### The catalytic activity of caspase-11 is required for the modulation of cofilin phosphorylation status

**Figure 8:**
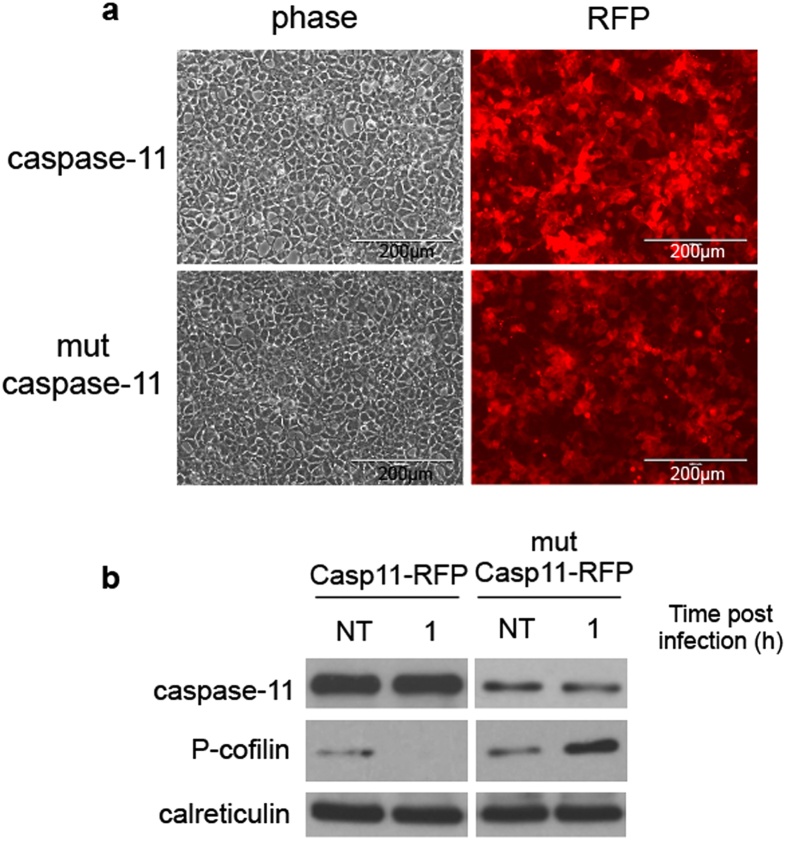
The dephosphorylation of cofilin is dependent on enzymatic activity of caspase-11. Immunoblot analysis of lysates of HEK293 cells transduced with lentiviral construct (**a**) containing enzymatically active and inactive (mut) caspase-11 red fluorescent plasmid after 1 h infection with *Legionella* with phospho-cofilin (P-cofilin) antibody (**b**). Blots are representative of three independent experiments.

Caspases, initially expressed as inactive zymogens, are activated by proteolytic processing in response to infection or insult stimuli^43^,^44^. After activation, caspases promote a myriad of downstream effects such as: production of inflammatory cytokines, restriction of intracellular bacteria, cell repair, and survival^33^. To determine if the enzymatic activity of caspase-11 is required for the modulation of cofilin phosphorylation, and hence actin polymerization, HEK293 cells were transduced with lentiviruses that contained either WT or a catalytically inactive (mut) caspase-11 tagged with a red fluorescent protein (RFP) (Fig. 8a). Cells stably expressing WT or mut-caspase-11 were infected with *Legionella* and the phosphorylation state of cofilin was assessed. HEK293 cells expressing caspase-11 or mut-caspase-11 exhibited disparate phospho-cofilin trends. Similar to WT macrophages, HEK293 cells expressing functional caspase-11 dephosphorylated cofilin upon *Legionella* infection. On the other hand, HEK293 cells with mut-caspase-11 failed to do so and instead the phosphorylation of cofilin was increased (Fig. 8b). Therefore, our data indicate that enzymatic activity of caspase-11 is essential for the dephosphorylation of cofilin during *Legionella* infection.

## Discussion

Much of the recent work in innate immunity detailing the functions of caspases has focused on their role in cytokine production and cell death with less consideration on emerging ancillary functions^44^,^45^. Recently, studies implicating alternative functions for caspases have come to light, including: unconventional protein secretion, lipid metabolism, membrane repair, phagosome acidification, and restriction of bacterial pathogens^28^,^46^,^47^,^48^. Caspases have been linked to the actin cytoskeleton in context of apoptosis^5^,^49^,^50^ and regulators and stabilizers of actin filaments have been found to be substrates of initiator and executioner caspases during apoptotic events^51^. However, whether caspases modulate the actin machinery independently of cell death is still unclear. To better understand the role of caspase-11 and caspase-1 in vesicular trafficking, we focused on physiological doses of *Legionella* infection and early trafficking events that mimic early infection stages. Hence, our data describe non-pyroptotic consequences of caspase activation.

The inducible caspase-11, like other caspases, executes apoptotic functions when strongly induced and activated and elicits non-apoptotic roles when moderately activated^7^,^28^,^51^,^52^,^53^. Reports have indicated that activation of caspase-11 leads to cell death in response to high doses of *Legionella*^35^,^52^,^54^,^55^. Conversely, using different bacterial strains and opsonization, Zamboni’s group nicely described how caspase-1 but not caspase-11 is required for cell death in response to *Legionella* infection^33^. The elegant work by Isberg group showed that low, physiological doses of *Legionella* actually upregulate anti-apoptotic genes, positively controlled by the transcriptional regulator nuclear factor kappa-light-chain-enhancer of activated B cells (NF-κB) thus, extending host cell survival^56^. Similarly, work from Nunez group demonstrated that caspase-1 promotes the trafficking of *Legionella* vacuole and increases bacterial clearance irrespective of cell death^17^.

In our study, using physiological doses of infection, we found that the death of WT and Casp-11^−/−^ macrophages is comparable, similar to that of Zamboni’s work^33^. Yet, *Legionella* survival *in vivo* and *in vitro* was significantly increased in *Casp-11*^−/−^ mice and their derived macrophages^14^. Accordingly, we found that at low infection levels, in WT cells, caspase-11 promotes the fusion of the *Legionella*-containing vacuole (LCV) with lysosomes leading to bacterial degradation, whereas in macrophages lacking caspase-11, the trafficking of the LCV is stalled and the pathogen escapes degradation^14^. On the other hand, in Casp-1^−/−^ macrophages, the LCV also escapes fusion with the lysosome while the host cell survives Fig. 1,2c,d. Therefore, according to our work and others in the field, it is clear that the intracellular survival of *Legionella* is not solely dictated by the death of the host cell (Table 1).

Here we report that resting WT macrophages exhibit uniform distribution of both F- and G-actin moieties, whereas Casp-11^−/−^ and Casp-1^−/−^Casp-11^Tg^ macrophages demonstrate F-actin accumulation at the cell periphery. Diverse distributions of F- and G-actin in uninfected WT, Casp-11^−/−^, and Casp-1^−/−^Casp-11^Tg^ did not affect bacterial uptake since macrophages harbored similar numbers of *Legionella* or *E. coli* at 1 hour post infection. Interestingly, caspase-11 is required for the phosphorylation of cofilin, while caspase-1 is essential for its dephosphorylation upon *Legionella* infection. Like Casp-1^−/−^Casp-11^Tg^ macrophages, macrophages lacking Nlrc4 or Naip5 also failed to dephosphorylate cofilin, confirming the contribution of these NLRs to the actin machinery upstream of caspase-1.

Notably, WT cells displayed dynamic changes in the F/G ratio during infection. However, vacuoles containing non-pathogenic bacteria such as *E. coli* traffic to the lysosome independently of any changes in the F/G ratio and irrespective of caspase-1 and caspase-11. The mechanism by which caspases recognize vacuoles that contain pathogenic organisms is unknown. Various reports have indicated that caspase-11 itself is a sensor for the bacterial factor lipopolysaccharide (LPS), in models where LPS is delivered to the cytoplasm or in cases where pathogens escape the intracellular vacuole^57^,^58^,^59^. Notably, *Legionella* LPS mutants still dephosphorylate cofilin in WT cells (data not shown). Convergence of caspase-11 and caspase-1 on cofilin is dependent on bacterial flagellin, as WT macrophages infected with either a flagellin (*flaA*^*−*^) or T4SS, (*dotA*^−^) mutant failed to dephosphorylate cofilin. These results indicate that access of flagellin to the cytosol is essential for the activation of caspase-11 and caspase-1 to alter the activation of cofilin.

**Figure 9:**
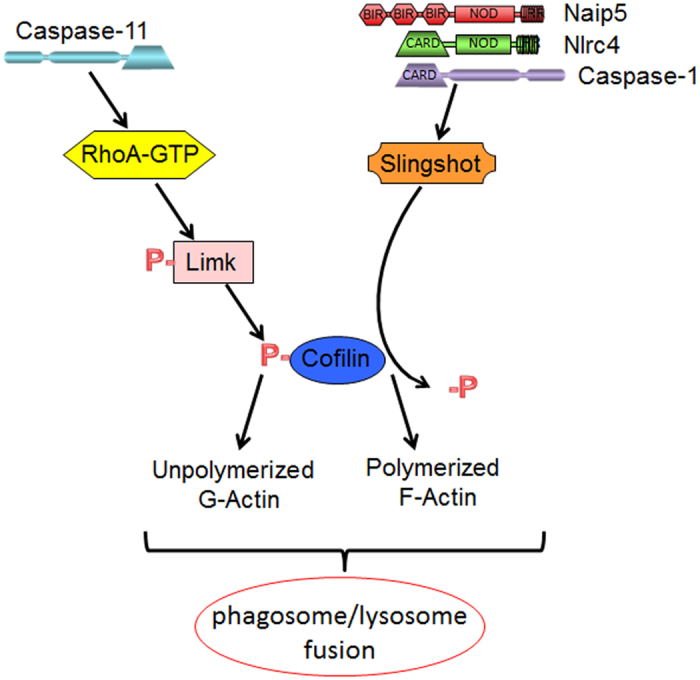
Caspase-11 is required for the activation of RhoA GTPase, while caspase-1 is essential for Slingshot phosphorylation. Accordingly, caspase-11 promotes the phosphorylation of cofilin and actin depolymerization, whereas caspase-1 promotes the dephosphorylation of cofilin and actin polymerization. The dynamics of F/G actin ratio facilitates the fusion of the *Legionella* vacuole with lysosomes.

Regulation of cofilin phosphorylation is mediated by upstream kinases and phosphatases (Fig. 9). These players are differentially phosphorylated in order to be activated. The molecular GTPases; RhoA, Rac, and Cdc42 promote downstream signaling to phosphorylate and activate Limk, whereas ATP, histamine, calcineurin, and λ phosphatase dephosphorylate Slingshot to promote its activation^5^,^6^,^41^,^60^,^61^,^62^. Casp-11^−/−^ exhibits defects in Rho-GTPase activation while Rho activity was similar in WT and Casp-1^−/−^Casp-11^Tg^ cells. On the other hand, dephosphorylation (activation) of the Slingshot phosphatase does not occur in cells lacking caspase-1 but proceeds efficiently in WT and Casp-11^−/−^ macrophages during *Legionella* infection. These data demonstrate that both caspases modulate cofilin phosphorylation in an opposing mode by differentially regulating upstream RhoA and Slingshot (Fig. 9). This intricate balance between phosphorylation and dephosphorylation of actin regulators is essential for vesicular trafficking to occur since it modulates the amount of polymerized and depolymerized actin.

Together our findings demonstrate for the first time the opposing effects of caspase-11and caspase-1 on the activation of an actin-associated factor. On the other hand, Yuan’s group has linked caspase-11 activity to cofilin modulation, via actin interacting protein 1 (Aip1), to promote lymphocyte migration^63^. More recently, it was found that Casp-11^−/−^ T cells migrate less efficiently into lymphoid tissues^64^. Likewise, Nlrc4 has been implicated in mediating actin polymerization, promoting cell rigidity and preventing cell migration^65^,^66^. Whether this recent Nlrc4 function is mediated through caspase-1 or caspase-11 remains to be determined.

The importance of the cytoskeleton in host defense is supported by the fact that *Legionella* devotes several molecules secreted through its T4SS that alter the cytoskeletal network^2^,^67^,^68^,^69^,^70^. Purification and proteomic analysis of pathogen-containing vacuoles identified the members of the actin machinery on the vacuolar membrane^71^. The involvement of actin in bacterial pathogenesis has been previously reported without description of a clear mechanism. Studies have demonstrated that increased actin polymerization during *S. typhimurium, L. donovani*, or *Mycobacteria* spp. prevents the fusion of the lysosome^37^,^65^,^72^,^73^,^74^. Therefore, the strategy of specific bacteria to modulate the cytoskeleton is an efficient tactic used by professional intracellular organisms in order to subvert restrictive vesicle trafficking.

These data raise the intriguing possibility that host cells activate caspases to promote cellular immunity by engaging their alternative non-apoptotic functions to control intracellular replication of pathogenic microbes.

## Methods

### Bacterial Strains

*Legionella pneumophila* (*Legionella*) strain Lp02 is a streptomycin resistant (Sm^r^) thymine-auxotroph derivative of Philadelphia-1. The *flaA*^*−*^ and T4SS (*dotA*^−^) mutants have been previously described^75^. Lp02 and *dotA*^−^ were complemented with a plasmid for green fluorescent protein (GFP). All *in vitro* infections were performed at an MOI of 0.5, unless otherwise stated, and were performed in the absence of thymidine, ferric nitrate, and L-cysteine to restrict extracellular growth. Non-fluorescent *Escherichia coli* (*E. coli*) strain BL21DE3 was grown overnight to Log phase as previously described^14^,^22^.

### Mice

Wild-type (restrictive) C57BL/6 (B6) were purchased from The Jackson Laboratory (Bar Harbor ME). Casp-11^−/−^ mice were generously donated by Dr. Yuan at Harvard Medical School^76^. Casp-1^−/−^/Casp-11^Tg^ mice were a gift from Dr. Vishva Dixit at Genentech^38^. Naip5^−/−^ mice were from Dr. Russell Vance at University of California Berkeley^24^. All mice were housed in a pathogen-free facility and experiments were performed with approval and in accordance with regulations and guidelines from the Institutional Animal Care and Use Committee (IACUC) at The Ohio State University (Columbus OH).

### Cell culture

Bone marrow-derived macrophages (BMDMs) were cultured as previously described^77^. Construction of red fluorescent caspase-11 and mutant caspase-11 using the pLenti6/V5 TOPO vector (Invitrogen Life Technologies) was previously described^78^. To generate HEK293 cells stably expressing red fluorescent caspase-11 and mutant caspase-11. Cells were transduced with lentivirus at an MOI of 1 in the presence of 6 μg/mL polybrene for 8–16 hrs. Afterwards, cell culture media was replaced and cells were selected with blasticidin (5 μg/mL) for 10 days resulting in a homogeneous yield of stably transduced cell line.

### Immunoblotting

Cell lysates were prepared with an isotonic buffer (10 mM HEPES, 5 mM MgCl_2_, 1 mM EGTA, 142 mM KCl with NP-40), in the presence of complete, EDTA-free, protease inhibitor cocktail (Roche). Samples were clarified, denatured with SDS buffer, and boiled. Proteins were separated by SDS-PAGE and then transferred to a polyvinylidene fluoride (PVDF) membrane (Biorad). Blots were probed with antibodies against caspase-11 (Sigma), caspase-1 (Genentech), phospho-cofilin, cofilin, Rac/Cdc42, and phospho-Rac (Cell Signaling), actin (Abcam), calreticulin (Stressgen). Detection was achieved using appropriate secondary antibodies conjugated with horseradish peroxidase (HRP), as previously described^12^,^14^,^22^,^79^.

### Confocal Microscopy

F- and G-actin were visualized from *Legionella*-infected macrophages using Alexa Fluor® 568 phalloidin and Alexa Fluor® 488 DNAse I (Molecular Probes) and for studies examining colocalization of GFP-expressing bacteria (*Legionella* and *E. coli*) with the lysosome, Lysotracker™ red was used to stain acidic vesicles of infected BMDMs, as previously described^14^. To examine cell death, after infection macrophages were stained with 4 uM ethidium homodimer-1 (EthD-1) from the LIVE/DEAD^®^ viability/cytotoxicity kit (Molecular Probes), according to manufacturer’s instructions. Images were captured using laser scanning confocal fluorescence microscope with a 60X objective (Olympus Fluoview FV10i).

### F/G actin Assay in Live cells

Amounts of G- and F-actin were assessed from lysates of WT, *Casp*-11^−/−^, and *Casp*-1^−/−^*Casp*-11^Tg^ according to the manufacturer’s recommendations (BK037, Cytoskeleton). Briefly, macrophages were infected and lysed with F-actin stabilization buffer. Lysates were then ultracentrifuged and cytoplasmic fractions were separated and run out on an SDS-PAGE gel. Antibodies specific for actin were then used to visualize amounts of F- and G-actin.

### RhoA and Rac GTPase Activity

Lysates from *Legionella*-infected macrophages were assayed for Rac1 and RhoA GTPase activity by G-LISA assay according to manufacturers recommendations (RhoA, BK124 and Rac, BK128; Cytoskeleton). Briefly, cell lysates were incubated on a Rac1 or RhoA affinity plate and then this colorimetric assay was developed with HRP detection reagent. Samples were read on a Spectra Max M2 plate reader (Molecular Devices) and results were expressed as absorbance at 490 nm.

### Statistical Analysis

Data were analyzed using GraphPad Prism software. Data in figures are presented as mean averages of at least 3 independent experiments and error bars represent SEM. Comparisons of groups for statistical significance were analyzed with Student’s two-tailed t-test. p value ≤0.05 was considered significant.

## Additional Information

**How to cite this article**: Caution, K. *et al.* Caspase-11 and caspase-1 differentially modulate actin polymerization via RhoA and Slingshot proteins to promote bacterial clearance. *Sci. Rep.* **5**, 18479; doi: 10.1038/srep18479 (2015).

## Figures and Tables

**Table 1:** Summary of caspase function during *Legionella* infection and outcomes.

	WT	Caspase-11^−/−^	Casp-1^−/−^Casp11^Tg^
Phagosome-Lysosome fusion	Efficient	Reduced	Reduced
Cell death	Efficient	Efficient	Reduced
Intracellular bacterial growth	Reduced	Increased	Increased
